# Exosomes in Lung Cancer: Actors and Heralds of Tumor Development

**DOI:** 10.3390/cancers13174330

**Published:** 2021-08-27

**Authors:** Amaia Sandúa, Estibaliz Alegre, Álvaro González

**Affiliations:** 1 Service of Biochemistry, Clínica Universidad de Navarra, 31008 Pamplona, Spain; asandua@unav.es (A.S.); ealegre@unav.es (E.A.); 2 Navarra Health Research Institute, IdiSNA, 31008 Pamplona, Spain

**Keywords:** lung cancer, biomarker, exosomes, isolation liquid biopsy, microvesicles

## Abstract

**Simple Summary:**

Lung cancer is a leading cause of cancer-related death worldwide and in most cases, detection is usually late and treatment resistance is frequent. For that reason, it is necessary to find biomarkers that could improve the diagnosis and disease management. Exosomes are a type of microvesicles secreted by tumor cells to the medium, with important functions in tumor development. Their analysis can be of utility in diagnosis, including early diagnosis, prognosis, treatment election or follow-up. However, isolation and analysis are cumbersome and can affect the subsequent data information. In this review, we will discuss the recent advances in the role of exosomes in lung cancer and their utility as liquid biopsy, with special attention to isolating methods.

**Abstract:**

Lung cancer is a leading cause of cancer-related death worldwide and in most cases, diagnosis is reached when the tumor has already spread and prognosis is quite poor. For that reason, the research for new biomarkers that could improve early diagnosis and its management is essential. Exosomes are microvesicles actively secreted by cells, especially by tumor cells, hauling molecules that mimic molecules of the producing cells. There are multiple methods for exosome isolation and analysis, although not standardized, and cancer exosomes from biological fluids are especially difficult to study. Exosomes’ cargo proteins, RNA, and DNA participate in the communication between cells, favoring lung cancer development by delivering signals for growth, metastasis, epithelial mesenchymal transition, angiogenesis, immunosuppression and even drug resistance. Exosome analysis can be useful as a type of liquid biopsy in the diagnosis, prognosis and follow-up of lung cancer. In this review, we will discuss recent advances in the role of exosomes in lung cancer and their utility as liquid biopsy, with special attention to isolating methods.

## 1. Lung Cancer and Exosomes

Lung cancer is a leading cause of cancer-related death worldwide with smoking being the main risk factor for this disease [1]. This cancer can be divided into two types of histology, small-cell lung cancer (SCLC), that comprises 15% of the cases, and non-small cell lung cancer (NSCLC), about 85% of all cases, of which adenocarcinoma and squamous cell carcinoma are the most common subtypes [2]. About 75% of patients are diagnosed at locally advanced or even metastatic stage, when the prognosis is considered grim with a 5-year survival rate of only 15%. Thus, lung cancer treatment would greatly benefit from early detection, as a delayed diagnosis will increase mortality risk.

The understanding of the molecular bases of lung cancer with the discovery of driver mutations, such as in epidermal growth factor receptor (*EGFR*) gene in lung adenocarcinoma [3], and the identification of immune checkpoints that regulate the tumor immune response [4] have allowed the development of new therapies. As a result, in recent years important progresses were made in the treatment with EGFR inhibitors, anaplastic lymphoma kinase inhibitors, or immune checkpoints inhibitors [3,4,5]. However, as in other malignancies, lung cancer is composed of different cell populations with varied molecular alterations resulting in tumor and microenvironment heterogeneity [6]. In fact, the initial predominant targetable alterations can become less abundant during the course of the disease due to the selection of resistant sub-clones. The identification of these molecular characteristics during the evolution of the disease is of paramount importance in order to develop an efficient therapeutic strategy suitable to each clinical situation. 

Tumor biopsy is the gold standard diagnostic procedure for histologic and molecular analysis. However, it is not always feasible due to difficult access to the lesions, and neither performing repeated biopsies during the course of the disease due to the procedure invasiveness. Additionally, biopsy may not reflect tumor heterogeneity. For that reason, it is necessary to find biomarkers with enough sensitivity and specificity for early diagnosis and for a close monitoring of the disease, helping in the choice of the best therapy for a personalized medicine. An alternative option to tissue biopsy is to perform the analysis in liquid biopsy, where repeated sampling can be easily performed [7]. Liquid biopsy in cancer consists of the analysis of three types of tumor-derived material in biological fluids: circulating tumor cells (CTCs), tumor-derived extracellular vesicles (EV), mainly exosomes, and circulating tumor DNA (ctDNA) [8]. Of them, ctDNA has attracted the most attention and several guidelines already include its analysis for the management of NSCLC [9,10]. Furthermore, some of these treatments mentioned before have already been allowed in patients with actionable mutations even when detected only in ctDNA [11].

Exosomes are spherical virus-size microvesicles with a density of 1.13–1.21 g/mL that participate in local and systemic intercellular communication transferring bioactive molecules between cells (reviewed in [12]). In the case of tumor-derived exosomes, they contribute to creating a favorable environment for tumor progression [13]. Consequently, tumor-derived exosomes play an important role in tumor development. Therefore, their analysis can help to gain a deeper knowledge of tumor biology and they can even be targets for drug therapy or delivery [14]. In addition, exosomes are very attractive due to their potential role as cancer biomarkers that could improve the management of cancer patients in general, and specifically, in lung cancer patients. In this review, we will summarize the role of exosomes in lung cancer development and their role as biomarkers of diagnosis, prognosis and even treatment election and follow-up.

## 2. Exosome Biogenesis and Structure

**Figure 1:**
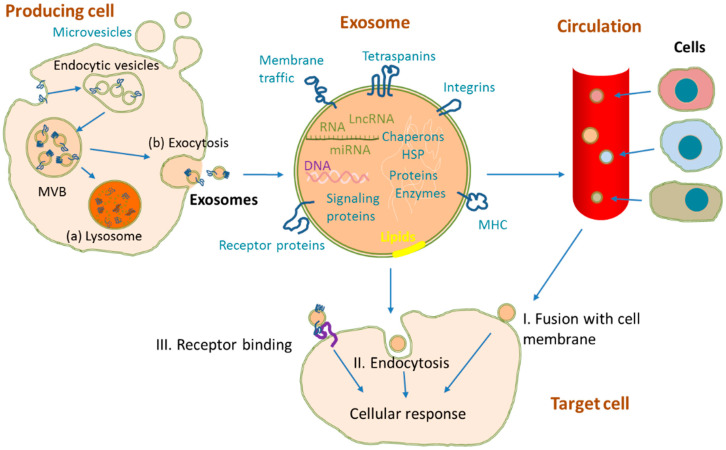
Exosome biogenesis, release and uptake from other cells. Exosomes are formed by initial inward budding of plasma membrane and the formation on endocytic vesicles. Endosomal vesicles then form multivesicular bodies (MVB), which can either be degraded in lysosomes (a) or fused with the plasmatic membrane releasing exosomes to the medium (b). Other microvesicles can be formed and shed by simple outward budding of the cell membrane. During exosome formation, there is a selective incorporation of RNA, DNA, proteins and lipids, many of them characteristic of the producing cell. Released exosomes can reach circulation and interact with the target cell by fusion with either the cell membrane (I), endocytosis (II), receptor binding (III), or in combination, inducing intracellular signals.

Cells release several types of microvesicles to the medium that differ in size, cellular origin and cargo: exosomes (50–200 nm), ectosomes (100–1000 nm) and apoptotic bodies (500–5000 nm) [15,16]. Exosome biogenesis initiates with the formation of the multivesicular bodies (MVB) containing many intraluminal vesicles formed by invagination of the endosomal membrane [15,16] (Figure 1). During this process, different materials from the parent cell, such as DNA, mRNAs, microRNAs (miRNAs), non-coding RNAs, lipids and proteins are selectively and actively incorporated into them [17]. Their release to the medium occurs through the fusion of multivesicular bodies with plasmatic membrane [16]. All cells can actively secrete exosomes, but it seems to be especially abundant in the case of tumor cells, with an estimation of 20,000 vesicles in 48 h by a single cancer cell [18]. Low oxygen tension and the resulting acidity due to increased glycolysis, typical conditions found in the tumoral microenvironment, favor the secretion of exosomes by cancer cells [19]. Increased secretion of tumor derived exosomes has been observed during the process of cancer development [20]. These exosomes can access circulation, where they have a short half-life and are cleared from blood in 6 h [21]. For example, Rabinowits et al. [22] found that plasmatic exosome levels were higher in lung adenocarcinoma patients compared to controls, probably due to alterations in cellular physiology. 

Exosome membrane is a lipid bilayer especially enriched in lipid rafts, such as those of cholesterol, sphingomyelin and ceramide, which makes these microvesicles very stable and protected from degradative processes in the extracellular space [23]. Several thousand exosomal molecules have already been documented in different databases such as Exocarta database (http://www.exocarta.org, accessed on 7 July 2021). Surface proteins include tetraspanins (CD9, CD63 and CD81), integrins and adhesion molecules or ligands that can interact with specific receptors or cells. Other proteins included in exosomes are heat-shock proteins (HSP60, HSP70, etc.) as well as others involved in membrane transport and fusion (RAB5b, flotillin, annexins, etc.) or in MVB biogenesis (ALG2-interacting protein X, ALIX, and tumor susceptibility gene 101 protein, TSG101). Some of the proteins carried by exosomes are characteristic of the producing cells and can help to identify exosomal origin. For example, trophoblast exosomes can be identified by HLA-G transported in them [24], immune cells exosomes transport MHC I and II molecules [25] or T lymphocytes exosomes carry CD3 antigen [26]. Similarly, exosomal nucleic acid content is related to the type of producing cell in addition to the sorting process. Therefore, all this complex exosome composition helps to explain their many different roles in intercellular communication, and their potential utility as liquid biopsy.

## 3. Exosome Isolation and Identification

Exosomes have been obtained from different biological fluids, such as serum, plasma, urine, cerebrospinal fluid or exudates [27,28,29,30]. Exosome isolation methods are mainly based on their physicochemical properties, such as size or density, or their biological characteristics and molecules expressed in their surface [31,32]. These methods differ in efficiency, purity, and even in their capability to select exosome subpopulations [33,34]. A summary of isolation methods can be found in Table 1 including some of the commercial kits already available.

Within the methods based on physicochemical characteristics, ultracentrifugation procedures are the most widely and traditionally used to isolate exosomes, but isolated vesicles purity is low as other particles or protein aggregates can also sediment with them [72]. Besides, there are other important drawbacks such as its cost, the long and complex process, the difficulty to scale-up the number of samples, and to be used in clinical settings. Other methods are based on particle size, being the most common ultrafiltration and size exclusion chromatography [34,54]. Precipitation methods are based on the change of either their solubility, aggregate formation or both, after the addition of polyethylene glycol, protamine or sodium acetate [33,65]. Compared to ultracentrifugation procedures, these methods are less time consuming, do not require large volumes of biological samples or special equipment, and can be scalable. However, they lack specificity and isolated exosomes can also be accompanied by impurities. Another more specific method, but quite costly, is based on the interaction of antibodies with specific molecules at the exosome surface such as CD81, CD63 or CD9 [31,64]. When using immunocapture assays it should be considered that their efficiency depends on the number of exosomes, the density of antigen per particle and the antibody affinity for the exposed epitopes. In fact, not all antibodies that work properly with free molecules are suitable for exosome capture, as the target epitope might not be accessible due to the orientation or the folding of the protein in the exosome [73]. Another important issue is that exosomes can easily adhere to working material surfaces, with the consequent risk of either losing interesting exosomes, high background signal or both [13]. Finally, some protocols combine successive isolation and purification methods, such as polymer precipitation and immunoaffinity purification, rendering a fairly pure population of exosomes [33,74]. 

Plasma is one of the most complex fluids and isolated exosomes from it can be contaminated with particle aggregates, plasma proteins, such as albumin or fibrinogen, or with similar sized lipoproteins [33,75]. These contaminants can further affect functional and analytical studies. For example, lipoproteins may carry miRNA that could interfere when studying these molecules in exosomes [76]. Furthermore, proteomic analysis or even immunological analysis are prone to provide biased results due to these contaminants [33].

Although many data available are related to tumor exosomes from in vitro experiments, much less are related to tumor specific exosomes obtained from biological fluids. Blood usually contains high concentrations of exosomes, 10^8^–10^11^ per mL, but most of them derive from blood cells, and tumor exosomes usually account for only a small proportion of the total circulating exosomes, making their isolation cumbersome [77]. The use of antibodies against exposed tumor antigens at the exosome membrane can be used to purify cancer exosomes [74]. This procedure can turn out well when specific tumor antigens exist, such as prostate specific antigen (PSA) in the case of prostate cancer exosomes, but this is not the case in lung cancer. Tumor marker MUC1, although not specific, is highly expressed in lung cancer exosomes [78], so it could be an antigen for their selective isolation by immunoaffinity. Alternatively, the epithelial cell surface molecule (EpCAM) is a frequent surface biomarker targeted for plasma tumor exosome enrichment in different epithelial cancers, including lung cancer [22,79].

Unfortunately, there is a lack of consensus on the best method, or even a standardized procedure for either exosome extraction, purification or both [80]. Related to this, the exosome isolation method should be taken into account when designing procedures as it might affect the subsequent experimental results [33]. For example, while in some experiments, it is necessary to recover the maximal amount of vesicles, and structure preservation and high purity are not necessary, in others on the contrary, the purity is of utmost importance. Other important points to consider are the starting volume and the possibility of scaling up the method, depending on the number of samples to be analyzed.

Depending on the isolation method, it is even difficult to unequivocally distinguish exosomes from other subgroups of microvesicles from the point of view of their size, density, morphology, or even biomarkers because these properties overlap between the different subclasses of microvesicles. Extracellular vesicles (EV) differentiation is difficult once released, and for this reason the International Society for Extracellular Vesicles (ISEV) recommends the use of the term extracellular vesicles when exosomes are not completely characterized [80].

**Figure 2:**
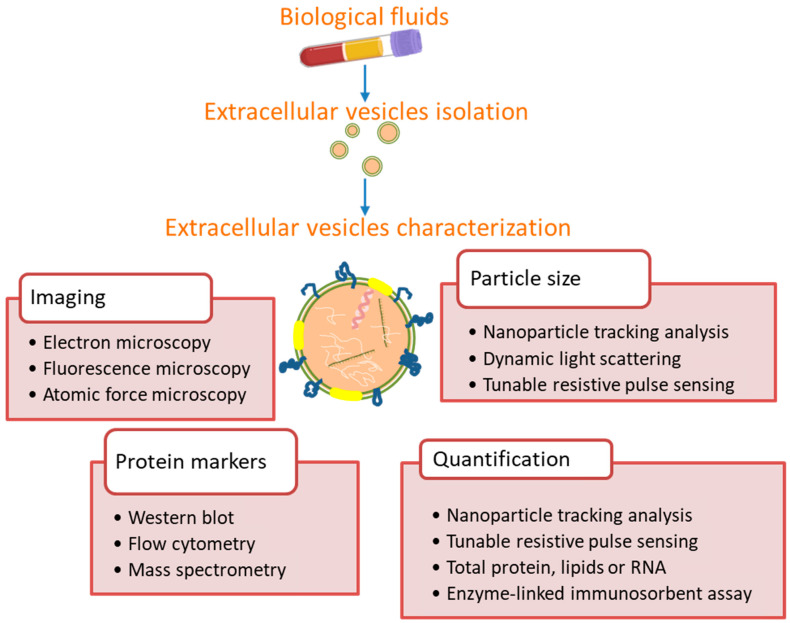
Methods commonly used for extracellular vesicles characterization from biological fluids. According the ISEV [80,81], extracellular vesicles should be described by protein markers and single particle characterization by imaging or sizing, and also quantified in relation to the source material.

Related to exosome characterization, the ISEV recommends using multiple complementary techniques to assess the results of extracellular vesicle-isolation methods (Figure 2) [80,81]. Exosomes can be identified by their size by transmission electron microscopy (TEM) or nanoparticle tracking analysis (NTA), which also allows measuring exosome concentration [82,83]. However, these methods do not distinguish exosomes from other nanoparticles with similar size. Specific exosomal proteins should be used as exosome biomarkers in combination with negative protein markers for better characterization. Membrane proteins, such as CD9, CD63 and CD81, or cytosolic proteins, such as TSG101, are frequent exosome markers detected by Western blot [80]. Purified EV should be quantified in terms of total particle number, protein or lipid content, in relation to the starting material. CD9, CD63 and CD81 have also been used for exosome quantification. Although they are expected to co-vary, CD63 can vary differently [84]. Furthermore, CD63 is under present in exosomes compared to cells, while CD81 can be up to 10-fold upregulated in exosomes [25].

As mentioned before, it is important to define the source material and the isolation method, as they can have an important impact on results, and characterize the extracellular microvesicles in order to know the purity and recovery [80,85]. For example, Macias et al. [33] showed that biomarker detection varied depending on the purification method used and there was no correlation in the concentrations of exosomes obtained with different procedures. For these reasons, we indicate isolation and characterization methods in the following tables showing the clinical utility of exosome biomarkers.

## 4. Exosome Function in Lung Cancer

**Figure 3:**
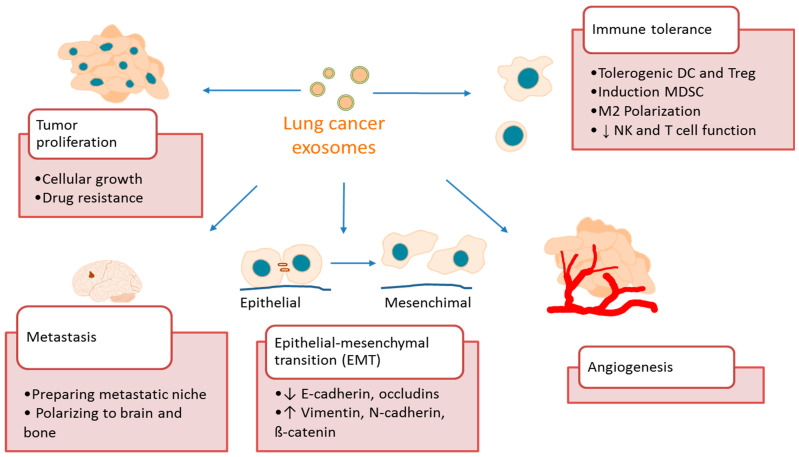
Role of exosomes in lung cancer. Tumor exosomes participate in key steps of cancer progression, such as tumor cell proliferation, epithelial-mesenchymal transition, tumor migration and metastases, induction of angiogenesis and immune tolerance.

Secreted exosomes can be captured by other cells by fusion with plasma membrane, endocytosis, micropinocytosis, phagocytosis or receptor-mediated specific binding [86]. Carried material interacts with target molecules in recipient cells triggering a cellular response: exosome mRNA can be translated into proteins [87], miRNA and lncRNA can modulate gene transcription and mRNA translation in target cells [88,89,90], and exosomal proteins can interact with receptors [91,92]. These bioactive molecules can induce tumor growth and modify cancer microenvironment, thus favoring cancer progression and metastasis [93]. More concretely, exosomes have been implicated in crucial steps of cancer development, such as tumor proliferation, epithelial-mesenchymal transition (EMT), tumor migration and metastases, induction of angiogenesis and immunosuppression (Figure 3). In the following paragraphs, we will discuss some examples of these mechanisms in lung cancer.

### 4.1. Exosomes Promote Lung Cancer Growth and Metastasis

Non-controlled cell proliferation is the basis of cancer growth and involves activation or altered expression of cell cycle genes and proteins. Tumor exosomes can carry molecules that can induce signals to stimulate tumor growth or even drive cell transformation [94]. miRNAs are the exosomal molecules that have probably been studied more extensively in relation to the different steps of the metastatic development. As an example, Wu et al. [95] showed that H1299 human lung adenocarcinoma cell line secretes miR-96-containing exosomes that inhibit the expression of LMO7, a tumor suppressor gene in lung cancer, and promote cell proliferation. Another study showed that A549 lung cancer adenocarcinoma cell line secretes exosomes engulfing miR-21 and miR-29a that bind Toll-like receptor TLR8 in immune cells, and trigger an NF-κB activation and secretion of inflammatory cytokines, thus favoring tumor growth and metastasis [88]. 

Mutations and gene amplifications of *EGFR* are important in NSCLC development and tyrosine kinase inhibitors (TKIs) have become a first-line therapy, although most patients relapse as drug resistance appears with time [2]. Different works revealed that exosomes could participate in the resistance to these drugs transferring miRNA or lncRNA from drug-resistant cancer cells to sensitive cells. For example, Zhang et al. [96] showed that gefitinib-resistant PC9 cells and their exosomes had high expression of miR-214. Those exosomes transferred miR-214 to sensitive PC9 cells that, as a result, acquired resistance. More recently, it was shown that exosomal transference of wild type EGFR promotes resistance to the TKI osimertinib by activating PI3K/AKT and MAPK signaling pathways [97]. Consequently, exosomes can also become therapeutic targets to overcome resistance development to these drugs.

An important step in tumor metastasis is EMT in which tumor cells lose their adherent characteristics of epithelial cells with decreasing expression of epithelial markers like E-cadherin and occludins, and acquire a mesenchymal phenotype with migratory and invasive capabilities, overexpressing mesenchymal markers like vimentin, N-cadherin o ß-catenin [98]. Different studies showed that exosomes participate in EMT in lung cancer transferring mesenchymal-induced signals and driving tumor cells to a more aggressive phenotype. For example, Rahman et al. showed that exosomes derived from highly metastatic lung cancer cells induced vimentin expression and EMT in HBE human bronchial epithelial cell line [99]. The highly metastatic lung cancer cell line SPC-A-1-BM and its exosomes were enriched in miR-499a-5p and by transferring this miRNA, these exosomes could increase the proliferation, migration and EMT via the mTOR pathway [100]. Cancer associated fibroblasts also secrete exosomes loaded with miR-210 that are uptaken by lung cancer cells inducing cell migration, proliferation, invasion abilities and EMT [89]. Finally, A549 cells, after TGF-β1-mediated EMT, release exosomes with cargo changes, both in protein and miRNA content, that induce further phenotypic changes via autocrine signaling [101].

An initial step for metastasis is the creation of a distant premetastatic niche with a favorable microenvironment where tumor cells can settle. Exosomes actively participate in this process, transporting active molecules in circulation that can modify target cells. In addition, through the carried molecules, particularly integrins, tumor exosomes can specifically target different organs or tissues and prepare the pre-metastatic niche [102]. Lewis lung carcinoma cell line produces exosomes containing miR-3473b, which once captured by lung fibroblasts cause NF-kB activation and inflammatory cytokines production, enhancing their intrapulmonary colonization [103]. Lung cancer commonly metastasizes to the brain and bone. Gang et al. [104] showed that lung cancer exosomes target brain microvascular endothelial cells inducing the release of Dkk-1 that provokes a displacement from M1 to a more pro-tumorigenic M2 phenotypic microglia. Subsequently, the metastatic lung cancer cells decrease Dkk-1 release removing the suppression on microglia that acquire a supportive phenotype. Furthermore, related to bone metastasis, Taverna et al. [91] observed that NSCLC exosomes contain amphiregulin, which binds EGFR in pre-osteoclasts activating the pathway that conducts to the expression of proteolytic enzymes initiating osteoclastic differentiation.

### 4.2. Exosomes Promote Lung Cancer Angiogenesis

Tumor growth is dependent on the blood supply with nutrients and oxygen, which requires the development of new vessels from the surrounding tissue. Tumor exosomes were shown to transport diverse molecules, especially miRNAs that once internalized by endothelial cells can induce neoangiogenesis. Hypoxia, which induces exosome release as we mentioned before, is very common in cancer and favors angiogenesis. For example, Hsu et al. [105] showed that lung cancer cells in hypoxic conditions secrete exosomes loaded with miR-23a, which once internalized in endothelial cells produces two effects in vasculature: first, it enhances angiogenesis by inhibiting prolyl hydroxylase 1 and 2, which produces accumulation of the hypoxia-inducible factor-1α (HIF-1 α); and secondly, it increases vascular permeability by inhibiting tight junction protein ZO-1 (zonula occludens 1 protein). Another study showed that exosomes from cigarette smoke extract-transformed human bronchial epithelial cells have high levels of miR-21 [94]. Exosomes transport this miR-21 into recipient normal human bronchial epithelial cells and induce elevated vascular endothelial growth factor (VEGF) levels promoting angiogenesis in human umbilical vein endothelial cells. Li et al. [106] observed that NSCLC cells overexpress leucine-rich-alpha2-glycoprotein 1 (LRG1), a protein that induces angiogenesis. Moreover, using the A549 cell line, they showed the release of exosomes loaded with LRG1, which induced, in endothelial cells, VEGF-A and angiopoietin-1 proangiogenic markers through a TGF-ß depended mechanism, and enhanced angiogenesis. 

### 4.3. Exosomes Promote Lung Cancer Immune Tolerance

One of the central issues for tumor development is immune evasion through the development of a tolerogenic microenvironment avoiding cellular killing, thereby facilitating tumor progression. Tumor exosomes’ cargo can suppress immune cell function by two mechanisms in the target cell: either indirectly reprogramming of cells to suppress immune functions in other cells, or directly blocking immune function. Membrane associated HSP72 from tumor-derived exosomes can bind TLR2 ligand on myeloid-derived suppressor cells inducing a STAT3-dependent immunosuppressive function [92]. Huang et al. [107] showed that lung cancer exosomes induce dendritic cells into a tolerogenic phenotype and, secondarily, naïve CD4+ T cells into tumor antigen-specific regulatory T cells, which could suppress the tumor antigen specific CD8+ T cells. Similarly, another study showed that lung tumor cells under hypoxia secrete microvesicles packed with the TGF-ß and miR-23a, which in turn, inhibit NK cell function decreasing the cell surface expression of the activating receptor NKG2D and the cytotoxic marker CD107a/LAMP1, respectively [108]. A common mechanism of immune evasion is the upregulation of immune checkpoints molecules, such as programmed death-ligand 1 (PD-L1), which interact with their corresponding receptor in T cell, suppressing the response. Cheng et al. [109] showed that metastatic melanoma, breast and lung cancer cells release extracellular vesicles, mostly exosomes, expressing surface PD-L1, whose levels increased after IFN-γ stimulation. Microvesicle PD-L1 binds PD-1 in the surface of CD8 T cells and suppresses the function, thus favoring tumor growth.

## 5. Exosomes as Biomarkers in Lung Cancer

Given the exosomal content, potential biomarkers comprise a wide variety of molecules including proteins and nucleic acids (Table 2, Table 3 and Table 4) Although initially many of the studies focused on proteins, in the last few years, miRNAs have attracted growing attention [110]. In many cases, utility does not rely on a single molecule but on a panel of them instead. Related to this and as it occurs with many other aspects of biological research, bioinformatics and big-data analysis have become key players, allowing management and evaluation of huge quantities of data in the search for the best markers.

### 5.1. Exosomal Proteins

Some of the studies in exosomal protein profiles have been performed using arrays that allow a multiplex analysis of exosomal proteins but without requiring a previous exosomes isolation [145]. For example, Sandfeld-Paulsen et al. [111] found that CD151, CD171 and tetraspanin 8 presented significant differences between controls and multiple lung cancer histological types, although the associated areas under ROC curve (AUC) for individual markers were quite limited with a maximum AUC of 0.68. Only when combining 10 markers in a panel could the AUC reach 0.76. Interestingly, some of these proteins, such as CD91, presented better diagnostic efficiency in other studies when combined with carcinoembryonic antigen (CEA) [112]. In fact, this and other studies combine exosomal markers with classical serological markers to achieve better diagnostic efficiencies. For instance, in another proteomic study of exosomal content, Niu et al. [113] detected higher levels of alpha-2-HS-glycoprotein (AHSG) and extracellular matrix protein 1 (ECM1) in serum samples from NSCLC patients when compared with healthy volunteers. In the case of AHSG, all cancer patients taken into account, the associated AUC was 0.736, which was reduced when selecting only early-stage patients. Regarding ECM1, both AUCs were similar and lower than that of AHSG. However, when AHSG was combined with CEA, the AUCs increased to 0.938 and 0.911, respectively, in total and early-stage NSCLC patients, substantially improving the efficiency of CEA alone. Meanwhile, Jakobsen et al. [84] performed this type of analysis with 37 antibodies. When combining CD81, CD63 and TAG72, the multivariate analysis showed an AUC of 0.758. Only when up to 30 proteins were included in the analysis, did the AUC reach a value of 0.830. A larger study performed with mass spectrometry identified 108 proteins with differential expression in exosomes between lung adenocarcinoma patients and healthy controls. Four of them, SRGN, TPM3, THBS1 and HUWE1, presented a combined AUC of 0.90 [114]. Another recent study identified CD5L as another potential biomarker [74]. This protein, an apoptosis inhibitor, was found to be overexpressed in both exosomes and cancer tissues and presented an AUC for lung cancer diagnosis of 0.943.

The term liquid biopsy usually refers to peripheral blood samples but other fluids’ analyses are also possible. Of particular interest is urine, which has the advantage of being less complex than plasma, although there are other issues to consider, such as the contamination from urine proteins or the time of collection. In fact, a recent article was published with methodological considerations from the Urine Task Force of the International Society for Extracellular Vesicles for exosome analysis in urine samples [146]. A proteomic study of lung cancer patients revealed high expression of LRG1 in urinary exosomes and lung tissue, suggesting that this protein can be a potential biomarker [115]. However, the specificity for lung cancer is expected to be low as other cancers also express LRG1 [147,148].

Regarding prognosis, Sandfeld-Paulsen et al. [116] observed an association between NY-ESO-1, EGFR, PLAP, EpCam and Alix from plasma exosomes with poor overall survival, although only NY-ESO-1 kept the association when Bonferroni correction was applied. 

*EGFR* gene evaluation has already become a key test in lung cancer management for prognosis and TKIs therapy election [149]. Additionally, EGFR protein evaluation in exosomes could also be of interest. For example, EGFR is present in 80% of exosomes purified from the lung cancer biopsies whereas in only 2% of exosomes from patients with chronic lung inflammation [107]. 

Another critical aspect of cancer management is therapy monitoring, and some exosomal biomarkers were also evaluated for this scope. For example, Yang et al. [117] showed that an increase in exosomal PD-L1 indicated response to treatment and better overall survival. Similarly, exosomal HSP70 is present in membranes of cancer-derived exosomes but not in exosomes from non-cancerous cells and correlate with HSP70 content within the tumor biopsies [118]. Moreover, HSP70 has been found useful not only in diagnosis and prognosis, but also in patients’ follow-up, with increasing levels in patients with disease progression and decreasing levels in those with partial or total response.

Table 2 shows a summary of recent clinical studies of the utility of exosomal proteins as biomarkers in lung cancer.

### 5.2. Exosomal miRNAs

As mentioned before, exosomal miRNAs have been implicated in lung cancer progression through multiple mechanisms including promotion of angiogenesis, vascular permeability and metastasis (reviewed in [150]). Some of these miRNAs were evaluated as biomarkers, mainly in diagnosis and prognosis (Table 3) [8]. In most cases, clinical utility does not rely on a specific miRNA but on a panel of multiple miRNAs. A combination of miR-151a-5p, miR-30a-3p, miR-200b-5p, miR-629, miR-100 and miR-154-3p achieved 96% sensitivity and 60% specificity in discriminating lung cancer from granuloma patients [119]. Wang et al. established a panel with four miRNAs (miR-9-3p, miR-205-5p, miR-210-5p, miR-1269a) that could discriminate NSCLC patients from healthy controls with an AUC of 0.91 (77% sensitivity and 89% specificity) [120]. Among them, miR-1269a presented the highest discriminatory capacity. Other candidates for being diagnostic biomarkers in early-stage NSCLC are miR-20b-5p and miR-3187-5p [124].

As exosomal proteins, miRNAs have also been combined with classical biomarkers of lung cancer. For example, miR-125b-5p’s usefulness has been evaluated as a diagnostic marker with discrete results when considering all stages (AUC = 0.700) and even lower when focusing on early-stage patients [121]. Its combination with CEA slightly improved CEA efficiency from 0.79 to 0.83. In addition, it could discriminate early versus advanced disease as well as the presence of lymph node and distant metastases. Another miRNA evaluated is miR-23b-3p that presented a diagnostic efficiency in ROC analysis of 0.915, much higher than those observed for classical serological markers such as CEA and CYFRA 21-1 [122]. Similarly, another study on miRNAs showed the utility in NSCLC diagnosis of let-7f-5p miRNA alone and in combination with CEA and CYFRA 21-1 [123]. Although diagnostic performance for NSCLC of the miRNA let-7f-5p is better compared to other conventional markers, it has a near perfect classification when combined with them, with an AUC of 0.981 (sensitivity of 94.7% and specificity of 93.3%).

In some cases, the utility does not rely on individual miRNAs’ levels but on the ratio between them instead, as in the miR-21/Let-7a ratio that clearly identified lung cancer patients from healthy volunteers (with sensitivity and specificity of 56% and 100%, respectively) and from those with pulmonary benign nodules (sensitivity and specificity were 56% and 82.6%, respectively) [125].

Exosomal miRNA analysis can help not only in diagnosis but also in histological classification. For example, Jin et al. [126] identified early-stage NSCLC patients with high sensitivity (80%) and greater specificity (92%) with a panel of miRNAs (let-7b-5p, let-7e-5p, miR-23a-3p and miR-486-5p). Moreover, these miRNAs allowed histological classification with miR-181b-5p and miR-361b-5p mainly being expressed in exosomes from adenocarcinoma patients, and miR-10b-5p and miR-320b in squamous cell carcinoma patients.

Multiple studies have analyzed both diagnostic and prognostic utility of exosomal miRNAs. For instance, Dejima et al. [127] evaluated miR-21 and miR-4257 and found, not only higher levels of both miRNAs in NSCLC patients, but also an association with clinical parameters such as tumor size and TNM stage in the case of miR-21, and with histological type, lymphatic invasion and TNM stage for miR-4257. Moreover, higher levels of both miR-21 and miR-4257 were associated with shorter disease-free survival.

Exosomal profile in pleural effusions can also be useful to detect lung malignancies. In fact, Lin et al. [128] observed that from 254 miRNAs detected in exosomes from pleural effusions, miR-205-5p and miR-200b could differentiate malignant effusions from those of pneumonia and tuberculosis patients. These two miRNAs were also included in a combination of exosomal miRNAs from peripheral blood (miR-429, miR-205, miR-200b, miR-203, miR-125b and miR-34b) that identified early-stage patients with a sensitivity of 85% and specificity of 74% [129]. More recently, Tamiya et al. [130] identified another pair of miRNAs, miR-182 and miR-210, which were able to identify malignant pleural effusions from benign ones with an AUC in ROC curves of 0.87 and 0.81, respectively.

Development of new and easy-to-use technological devices already allows the transfer of exosome research to clinical application. For example, a point-of-care device was recently developed to analyze salivary and urinary miR-205 [131], one of the miRNAs identified with diagnostic utility in some of the studies mentioned before.

About 20–40% of lung cancer patients develop bone metastasis with a negative impact in overall survival [151]. Yang et al. [132] identified three exosomal miRNAs with differential expression in NSCLC patients depending on whether they had bone metastasis or not. In the case of miR-574-5p, it was downregulated, while miR-328-3p and miR-423-3p were upregulated. All of them participate in the Wnt/β-catenin signaling pathway and thus can regulate metastasis development.

Exosomal miRNAs were also evaluated in the context of treatment election and follow-up. Regarding chemotherapy resistance, Yuwen et al. [133] observed that NSCLC patients with low expression of miR-146a-5p presented shorter progression-free survival. Moreover, the overexpression of miR-146a-5p could revert chemoresistance to cisplatin in A549 lung cancer cells by inhibiting autophagy. Regarding radiotherapy, Zheng et al. [134] showed that miR-96 can identify lung cancer patients with high efficiency (AUC = 0.97) but it also presents potential utility to identify radioresistant patients (AUC = 0.75). Another treatment option that has become a key strategy against lung cancer is immunotherapy, with PD-1/PD-L1 as one of the targeted checkpoints. Related to this, exosomes can also inform of the potential response to anti-PD-1 treatments. In a study from Peng et al. [135], a signature of three miRNAs from the miRNA-320 family could predict the efficacy of this type of therapy whereas downregulation of miR-125b-5p during treatment identifies patients in partial response.

### 5.3. Other Nucleic Acids

Although miRNAs are the most studied nucleic acids in exosomes, other molecules have also proved their utility as biomarkers in lung cancer patients (Table 4). In this way, Cao et al. [136] identified four mRNAs contained in exosomes that distinguished squamous carcinoma and adenocarcinoma. The combination of these mRNAs, tumor protein P63 (TP63), keratin 5 (KRT5), CEA cell adhesion molecule 6 (CEACAM6) and surfactant protein B (SFTPB), improved their histological classification capacity. Related to prognosis, exosomal eIF4E RNA was associated with TNM stage and the presence of metastases [137]. Furthermore, patients with higher levels presented shorter survival. 

RNA analysis allows the detection of *EML4-ALK* fusion that identifies patients that would develop resistance to EGFR inhibitors and would be susceptible to being treated with ALK inhibitors. Brinkmann et al. has observed that exosomal RNA can reflect the fusion transcript observed in tissue, becoming a potential alternative when tissue biopsies are not an option [152]. Moreover, exosomal mRNAs could also serve as biomarkers of immunotherapy efficacy. For instance, Del Re et al. [138] showed that patients receiving nivolumab or pembrolizumab with high baseline IFN-γ mRNA levels in exosomes had shorter progression-free survival than those with lower levels. Similarly, patients that progressed within three months, presented higher levels than those that responded or had disease stabilization.

Exosomal long non-coding RNAs (lncRNAs) were also evaluated as potential clinical biomarkers for lung cancer management. For example, in 2017, Zhang et al. [90], showed the utility of MALAT-1 as a diagnostic biomarker of NSCLC. Moreover, this lncRNA presented prognostic utility as it correlated with tumor stage and lymphatic metastases. Since then, multiple lncRNAs’ utility was proved not only in diagnosis and prognosis, but also as therapy targets (reviewed in [153]). More recently, linc01125 could distinguish NSCLC cases from disease-free and tuberculosis controls and correlated with an unfavorable overall survival [139]. Related to these lncRNAs, there is even a meta-analysis evaluating their diagnostic capacity [154]. 

Given the recent finding of circular RNA, there are far fewer studies assessing their role in lung cancer, but some of them have proved their utility as potential biomarkers. For instance, Li et al. showed that FLI1 exonic circular RNA (FECR) was increased in SCLC patients and it was correlated with the metastatic status [140]. In another study, circ_0014235 and circ_0025580 presented diagnostic utility to identify squamous cell carcinoma patients, and were strongly correlated with higher TNM stage and tumor size [141]. circRNA_0056616 also proved its capability to identify patients with lymph node metastasis [142]. Similarly, circSATB2 could also detect lung cancer metastasis [143]. Xian et al. showed the diagnostic utility of a panel comprising three circRNAs, circ_0047921, circ_0007761 and circ_0056285, to differentiate NSCLC patients from not only healthy controls but also from patients with other types of pulmonary diseases. The latter, also presented prognostic utility [144]. 

## 6. Conclusions

Exosomes are important players in lung cancer development participating in tumor aggressiveness such as in metastasis, with organ polarization to brain and bone, angiogenesis, immune escape and even drug resistance. Due to their size and capability to transfer molecules into target recipient cells, exosomes also postulate as potential drug delivery vehicles [14]. As the tumor exosomal cargo includes molecules from the releasing cells and can be detected in circulation, exosomes can serve as non-invasive biomarkers providing a potential alternative or at least, a complementary tool to conventional biopsy with additional advantages in the diagnosis, prognosis, therapy election and follow-up. Furthermore, the analysis of the complex composition of exosomes can provide a multianalyte approach that could give a dynamic insight into the tumor microenvironment, helping to provide a more precise and rapid medical intervention. This is important, as the implementation of different therapeutic strategies, using new cancer drugs discovered in the last years, needs appropriate biomarkers for guidance. In addition, exosome analysis could help in the screening and early detection of lung cancer, when patients have better prognosis [113,124], and some clinical trials are already addressing this issue (www.clinicaltrials.gov). However, exosome analysis has not been included in clinical guides yet, contrary to ctDNA where there are already clinical indications for its use as biomarker in lung cancer and some commercial kits are already available for mutations’ assessment [8,155]. One of the reasons is the lack of standardized protocols for exosome isolation and analysis, which impairs the implementation of their analysis in a routine clinical laboratory. Sophisticated technology, but also affordable and easy-to-use, for exosome analysis would also help to implement their use. In addition, studies are usually retrospective and with small cohorts and thus, more prospective studies with larger populations are needed. Finally, it is difficult to select the exosome biomarkers that correlated better with the clinical situation between different reported studies. Once these issues are solved, exosomes will probably be key participants in lung cancer management. To achieve this, it is necessary to develop more translational research and clinical trials before introducing exosomes in the management of lung cancer. 

## Figures and Tables

**Table 1:** Methods for exosome isolation.

Method	Isolation Principle	Assessment Parameters			Advantages	Disadvantages	Examples of Available Commercial Kits	References
		Time	Purity	Recovery				
Ultracentrifugation	Density by centrifugations at increasing speeds	+++	+	+	Isolation of large volumes, no addition of chemicals, no pretreatment needed, most used method	Time consuming, expensive equipment, low purity, low reproducibility, damage of vesicles		[27,29,30,31,32,35,36,37,38,39,40,41,42,43,44,45,46,47,48,49]
Density gradient ultracentrifugation	Density by centrifugations in a density gradient	+++	++	+	Effective in separation of EV from protein aggregates, high purity, no addition of chemicals	Time consuming, complex, low yields, fails to separate large vesicles with similar sedimentation rates	OptiPrep	[30,31,32,35,42,43,46,47]
Ultrafiltration	Size and molecular weight. Membranes with defined pore diameter or molecular weight cut offs	++	+	++	Simple and fast procedure, no special instrumentation, scalable	Clogging and trapping of vesicles on the filter, low yield, deformation of vesicles and lysis of exosomes, low purity	Amicon Ultra Centrifugal filters Vivaspin Centrifugal Concentrators	[30,43,44,50,51,52,53,54,55]
Hydrostatic filtration dialysis	Size. Diffusion of particles across a porous membrane at concentration gradient	+++	+	++	Simple, inexpensive, scalable, appropriate for diluted samples as urine	Selectivity of separation dependent on the cut-off, low purity		[29,56,57,58]
Size exclusion chromatography	Size. Small particles penetrate a porous stationary phase and elute at different rates	++	++	++	Preserves vesicles integrity and biological activity, high recovery and reproducibility	Low yield, might require concentration, difficulty in scaling	Exo-spin qEV Extracellular Vesicle Isolation	[27,33,34,39,43,44,47,52,53,59,60,61]
Asymmetric flow field-flow fractionation (AF4)	Size. Separation of particles in a channel with parabolic longitudinal flow combined with an external gradient	++	++	++	Possible EV subpopulation separation, possibility to couple to multidetection systems	Time consuming procedure, requires special equipment		[47,50,62,63]
Immunoaffinity	Specific binding between antigens expressed on the exosome surface and corresponding antibodies	++	+++	+	High purity and specificity, high selectivity, preservation of the activity of exosomal proteins, no protein contamination	Low yield, expensive, no scaling-up, EV cannot be readily eluted off the complexes with antibodies, antigenic epitopes might be blocked or masked	Dynabeads ExoFlow96 and 32 Exosome IP Kits ExoRNeasy Serum/Plasma Maxi Kit	[31,32,33,39,45,46,47,64]
Precipitation with polymers	Change in either the solubility, aggregate formation or both, after reagent addition	++	+	+++	High recovery, simple and fast procedure, no expensive equipment requirement, scalable	Low purity	ExoQuick Invitrogen Total Exosome Isolation Kit	[33,34,35,39,43,45,47,48,49,65]
Microfluidics technology	Separation according to size, external markers or innovative sorting mechanisms such as acoustic, electrophoretic or electromagnetic fields	++	+++	+++	High purity and recovery, efficiency, minimal sample volume and reagent consumption, fast, reduce cross-contamination	Cost, additional equipment and complexity of devices		[39,66,67,68,69,70,71]

Assessment parameters: +: short/low; ++: medium; +++: long/high.

**Table 2:** Studies on clinical utility of exosomal proteins as biomarkers in lung cancer.

Molecule	Sample	Number of Subjects	Isolation Methods	Characterization Methods	Utility	Comments	Authors
CD151, CD171, and tetraspanin 8	Plasma	336 LC + 126 C	EV array	-	Diagnosis	AUC calculated between LC and controls and when subdividing in AC, SCC and SCLC. NYESO1, HER2, EGFRvIII, SFTPD, Florilin1, CD142 and Mucin 16 also analyzed	Sandfeld-Paulsen et al. [111]
CD91 (+CEA)	Serum	Screening set: 10 C, 10 IP, 14 AC, 12 SCC Validation set: 54 C, 19 IP, 105 AC, 34 SCC	Immune-affinity for screening set ELISA with anti-CD9 in validation set	-	Diagnosis	Screening set: isolation by immune-affinity with anti-CD9 tips and proteomic study to identify CD9 Validation Set: ELISA with anti CD9 as capture antibody and anti-CD91 as detection antibody	Ueda et al. [112]
AHSG and ECM1	Serum	125 NSCLC + 46 C	Ultracentrifugation	TEM/NTA/WB	Diagnosis (including early stage)	Differentially expressed proteins identified by mass spectrometry	Niu et al. [113]
Panel of 30 proteins	Plasma	109 advanced NSCLC + 110 C	EV Array	-	Diagnosis	Array for 37 proteins	Jakobsen et al. [84]
SRGN, TPM3, THBS1 and HUWE1	Plasma	13 AC + 15 C	Density gradient	TEM/NTA/WB	Diagnosis	108 differentially expressed proteins identified by mass spectrometry	Vykoukal et al. [114]
CD5L, CLEC3B, ITIH4, SERFINF1, SAA4, SERFINC1, and C20ORF3	Serum	20 AC + 20 SCC + 20 SCLC + 20 C	Polyethylene glycol -based precipitation and immunoaffinity separation using antibodies against CD9, CD63, CD81, and EpCAM	TEM/NTA/DLS/WB	Diagnosis	Differentially expressed proteins identified by mass spectrometry; 55 confirmed by Western blot. CD5L highest AUC	Choi et al. [74]
LRG1	Urine	8 NSCLC + 10 C	Ultracentrifugation	TEM	Diagnosis	Differentially expressed proteins identified by mass spectrometry	Li et al. [115]
CD171 (1), NY-ESO-1 (2)	EDTA Plasma	276 NSCLC	EV array	-	Prognosis: (1) OS, (2) HR	Array for 49 proteins	Sandfeld-Paulsen et al. [116]
PD-L1	Plasma	33 NSCLC	Precipitation	TEM/NTA/WB	Prognosis: OS and PFS	Quantification with Simoa Bead Technology	Yang et al. [117]
HSP70	EDTA Plasma	20NSCLC+ 14 C + 10 BC	Ultracentrifugation	NTA/TEM	Diagnosis, prognosis (metastasis detection), monitoring	HSP70 barely detected in plasma. Exosomal HSP70 correlates with tissue analysis	Chanteloup et al. [118]

Abbreviations: AC: adenocarcinoma; AUC: area under ROC curve; BC: breast cancer; C: controls; CEA: carcinoembryonic antigen; CYFRA21-1: cytokeratin 19 fragment; DLS: dynamic light scattering; EDTA: ethylenediaminetetraacetic acid; EV: extracellular vesicles; HR: hazard ratio; IP: interstitial pneumonia; LC: lung cancer; NSCLC: non-small cell lung cancer; NTA: nanoparticle tracking analysis; OS: overall survival; P: pneumonia; PFS: progression-free survival; SCC: squamous carcinoma; SCLC: small cell lung cancer; T: tuberculosis; TEM: transmission electron microscopy; TNM: tumor-node-metastasis; WB: Western blot.

**Table 3:** Studies on clinical utility of exosomal miRNAs as biomarkers in lung cancer.

Molecule	Sample	Number of Subjects	Isolation Methods	Characterization Methods	Utility	Comments	Authors
(1) miR-378a, miR-379, miR-139-5p, and miR-200b-5p (2) miR-151a-5p, miR-30a-3p, miR-200b-5p, miR-629, miR-100, and miR-154-3p	Plasma	Screening set: 10 AC+ 10 LG + 10 C Validation set: 50 AC+ 30 LG + 25 C	Precipitation	-	(1) Diagnosis AC+ LG vs. C (2) Diagnosis AC vs. LG	Wide-range miRNAs analysis (742 microRNAs)	Cazzoli et al. [119]
miR-9-3p, miR-205-5p, miR-210-5p and miR-1269a	Serum	Training set: 74 NSCLC + 74 C Validation set: 73 NSCLC + 75 C	Precipitation	TEM/NTA/WB	Diagnosis	10 miRNAs to be analyzed were selected previously from TCGA database	Wang et al. [120]
miR-5684 (1) and miR-125b-5p (1, 2, 3) +CEA	Serum	330 NSCLC + 312 C	Ultracentrifugation	TEM/tunable resistive pulse sensing/WB	(1) Diagnosis, (2) Prognosis: Metastasis detection and survival, (3) therapy monitoring	22 miRNAs profiled by microarrays and verified by quantitative PCR	Zhang et al. [121]
miR-23b-3p + CEA + CYFRA21-1	Serum	80 NSCLC + 60 P + 30 C	Precipitation	TEM/NTA	Diagnosis Prognosis: tumor size, depth of invasion, liver metastasis and TNM stage	Quantification by RT-PCR. miRNA-39 was used as the external reference gene	Wang et al. [122]
let-7f-5p (1) miR-320a, miR-622 and let-7f-5p (2) + CEA and CYFRA21-1	Plasma	80 NSCLC + 30 C	Membrane affinity spin columns	-	(1) Diagnosis (2) Metastasis detection	miRNA array	Wang et al. [123]
miR-20b-5p and miR-3187-5p	Serum	276 NSCLC (104 stage I) + 282 C	Ultracentrifugation	TEM/NTA/WB	Diagnosis (including early stage)	miRNAs profiled by microarrays and verified by quantitative PCR	Zhang et al. [124]
miR-21/Let-7a ratio	Serum	75 NSCLC + 23 BPN + 18 PID +24 C	Precipitation	-	Diagnosis (including versus benign and inflammatory lung diseases)	Quantification by RT-PCR	Yang et al. [125]
let-7, miR-21, miR-24, and miR-486 (1) miR-181-5p, miR-30a-3p, miR-30e-3p, and miR-361-5p (2) miR-10b-5p, miR-15b-5p, and miR-320b (3)	Plasma	Testing set: stage I (16 AC + 10 SCC) + 12 C Validation set: stage I (10 AC + 10 SCC) + 30 C Symptomatic set 60	Ultracentrifugation + immune-affinity with anti-EpCAM beads	NTA/WB	(1) Diagnosis at early stage (2) Histological classification: AC (3) Histological classification: SCC	Small RNA profile with RNA NGS and subsequent confirmation with RT-PCR. Normalization with cel-miR-39	Jin et al. [126]
miR-4257 and miR-21	EDTA Plasma	Screening set: 6 NSCLC Validation set: 129 stage I + 34 stage II +32 stage III + 30 C	Ultracentrifugation	TEM	Histological classification Prognosis: TNM stage, tumor size, lymphatic invasion, disease-free survival	miRNA selected with an array in 6 NSCLC patients (3 with and 3 without recurrence)	Dejima et al. [127]
miR-205-5p and miR-200b	Pleural effusion	9 LC + 9 P + 9 T	Ultracentrifugation	TEM/NTA/WB	Diagnosis	Small RNA sequencing and subsequent confirmation with RT-PCR in 8 randomly chosen miRNAs	Lin et al. [128]
miR-429, miR-205, miR-200b, miR-203, miR-125b and miR-34b	Serum	Discovery set: 38 NSCLC + 16 COPD + 16 C Technical validation set: 16 NSCLC + 8 COPD + 6 C External validation set: 100 NSCLC + 58 C	Precipitation	-	Diagnosis (including early stage)	754 microRNAs screened with TaqMan Low Density Arrays. In the 10 miRNAs upregulated a technical validation was performed by RT-PCR. Global normalization was performed	Halvorsen et al. [129]
miR-182 and miR-210	Pleural effusion	41 AC + 15 BPE	Precipitation	-	Diagnosis	miR-21, miR-31, miR-182, and miR-210 analyzed by RT-PCR. Normalization with miR-16	Tamiya et al. [130]
miRNA-205	Urine and saliva	5 LC+ 5 C	Fe_3_O_4_@SiO_2_-aptamer nanoparticles	WB	Diagnosis	Development of a POCT device	Zhou et al. [131]
miR-574-5p and miR-328-3p and miR-423-3p	Plasma	30 NSCLC (16 with and 14 without bone metastasis) + 14 C	Ultracentrifugation	WB	Bone metastasis detection	Small RNA sequencing	Yang et al. [132]
miR-146a-5p	Serum	100 NSCLC with cisplatin-based chemotherapy	Precipitation	TEM/NTA/WB	Chemotherapy resistance Prognosis	Absolute miRNA levels quantify with RT-PCR with standard curves. Relative levels related to exosomal protein content	Yuwen et al. [133]
miR-1246 (1) and miR-96 (1,2,3)	Heparin Plasma	52 NSCLC (27 Radioresistant + 25 radiosensitive) + 45 C	Lipid nanoprobe	TEM/NTA/WB	(1) Diagnosis (2) Radioresistance detection (3) Prognosis: OS	miR-21, miR-1246, let-7g, miR-210, miR-214, and miR-96 analyzed by RT-PCR. Normalization with cel-miR-39	Zheng et al. [134]
hsa-miR-320d, hsa-miR-320c, and hsa-miR-320b	Plasma	5 NSCLC with partial response to PD-1/PD-L1 inhibitors + 4 with progression + 7 C	Ultracentrifugation	TEM	Response to PD-1/PD-L1 inhibitors	Small RNA profile with RNA NGS; 155 miRNAs differentially expressed versus controls	Peng et al. [135]

Abbreviations: AC: adenocarcinoma; BPE: benign pleural effusion; BPN: benign pulmonary nodules; C: controls; CEA: carcinoembryonic antigen; COPD: chronic obstructive pulmonary disease; CYFRA21-1: cytokeratin 19 fragment; EV: extracellular vesicles; LC: lung cancer; LG: lung granuloma; miRNA: microRNA; NGS: next generation sequencing; NSCLC: non-small cell lung cancer; NTA: nanoparticle tracking analysis; OS: overall survival; P: pneumonia; PFS: progression-free survival; PID: pulmonary inflammation diseases; RT-PCR: real-time polymerase chain reaction; SCC: squamous carcinoma; SCLC: small cell lung cancer; T: tuberculosis; TCGA: The Cancer Genome Atlas; TEM: transmission electron microscopy; TNM: tumor-node-metastasis; WB: Western blot.

**Table 4:** Studies on clinical utility of exosomal mRNAs, lncRNAs and circRNAs as biomarkers in lung cancer.

Molecule	Sample	Number of Subjects	Isolation Methods	Characterization Methods	Utility	Comments	Authors
TP63, KRT5, CEACAM6 and SFTPB mRNAs	Serum	54 AC + 16 SCC	Ultracentrifugation	TEM/NTA/WB	Histological classification	17 miRNAs to be analyzed were selected previously from TCGA database as differentially expressed between AC and SCC. ACTB and SLC25A6 were used as internal references	Cao et al. [136]
eIF4E RNA	Serum	99 NSCLC + 40 C	Precipitation	TEM/NTA/WB	Diagnosis Prognosis: stage, distant metastases, OS and PFS	eIF4E data extracted from TCGA database	Dong et al. [137]
PD-L1 (1) and IFN-γ (1,2) mRNA	EDTA Plasma	38 NSCLC	Membrane affinity spin columns	-	(1) Response to treatment (2) PFS	Quantification by ddPCR with ACTB as internal control	Del Re et al. [138]
MALAT-1	Serum	77 NSCLC + 30 C	Precipitation	TEM/NTA/WB	Diagnosis Prognosis (Lymph node metastasis, TNM stage)	Quantification by RT-PCR. GAPDH was used for normalization	Zhang et al. [90]
linc01125	Serum	277 NSCLC + 187 C + 5 P + 59 T + 58 COPD	Precipitation	-	Diagnosis Prognosis (stage, OS)	RNA-Seq for lncRNA profile and subsequent quantification of linc01125 by RT-PCR with spiked in controls	Xian et al. [139]
FECR	Serum	35 with limited SCLC and 26 with extensive SCLC +55 C	Affinity Chromatography	TEM/WB	Diagnosis Prognosis (survival) Response to chemotherapy	RT-PCR with β-actin as control	Li et al. [140]
circ_0014235 and circ_0025580	Plasma	30 SCC + 30 C	Precipitation	-	Diagnosis Prognosis (TNM stage and tumor size)	circRNA sequencing and confirmation with RT-PCR with GAPDH as internal control	Wang et al. [141]
circRNA_0056616	EDTA plasma	90 AC (42 with lymph node metastasis and 48 without)	Precipitation	TEM/WB	Lymph node metastasis predictor	RT-PCR. Normalization as Wang’s methods	He et al. [142]
circSATB2	Serum	83 NSCLC + 95 C	Ultracentrifugation	TEM/NTA/WB	Diagnosis Prognosis (metastasis detection)	RT-PCR. GAPDH and U6 were used as internal references and cel-miR-39 as an external reference	Zhang et al. [143]
circ_0047921, and circ_0007761 (1) circ_0056285 (1,2)	Serum	Screening set: 30 NSCLC + 45 C Training set: 120 NSCLC + 165 C Validation set 1: 62 NSCLC + 95 C Validation set 2: 63 NSCL + 58 COPD + 46 T	Precipitation	TEM/NTA/WB/FC	(1) Diagnosis (including early stage) (2) Prognosis: state of progression and lymph-node metastases	1701 circRNAs initially identified by RNA-seq, 17 of them were differentially expressed and 8 of them were validated by RT-PCR with GAPDH and ACTB as spiked-in controls	Xian et al. [144]

Abbreviations: AC: adenocarcinoma; C: controls; circRNA: circular RNA; COPD: chronic obstructive pulmonary disease; ddPCR: droplet digital PCR; EV: extracellular vesicles; FC: flow cytometry; LC: lung cancer; miRNA: microRNA; NSCLC: non-small cell lung cancer; NTA: nanoparticle tracking analysis; OS: overall survival; P: pneumonia; PFS: progression-free survival; RT-PCR: real-time polymerase chain reaction; SCC: squamous carcinoma; SCLC: small cell lung cancer; T: tuberculosis; TCGA: The Cancer Genome Atlas; TEM: transmission electron microscopy; TNM: tumor-node-metastasis; WB: Western blot.
